# Supplementary material for: Recovery of Natural Antioxidants from Onion Solid Waste via Pressurized Liquid Extraction: Encapsulation and Application into a Food System
Source: Foods. 2025 Oct 21;14(20):3583. doi: 10.3390/foods14203583 (PMC12564815; doi:10.3390/foods14203583)
Supplement: Supplementary file 1 [file foods-14-03583-s001.zip › foods-3907459-supplementary.pdf]

foods

MDPI

Supplementary Material

Recovery of Natural Antioxidants from Onion Solid Waste via Pressurized Liquid Extraction: Encapsulation and Application into a Food System

Eleni Bozinou [1,2], Nafsika-Thalia Georgiadou [2], Maria-Stella Chalastara [2], Ioannis Makrygiannis [2], Martha Mantiniotou [2], Vassilis Athanasiadis [2], Arhontoula Chatzilazarou [1,*] and Stavros I. Lalas [2,*]

[1] Department of Wine, Vine & Beverage Sciences, University of West Attica, Ag. Spyridonos Str., 12243 Athens, Greece; ebozinou@uniwa.gr (E.B.)

[2] Department of Food Science and Nutrition, University of Thessaly, Terma N. Temponera Street, 43100 Karditsa, Greece; nageorgiadou@uth.gr (N.-T.G.); mchalastara@uth.gr (M.-S.C.); ioanmakr1@uth.gr (I.M.); mmantiniotou@uth.gr (M.M.); vaathanasiadis@uth.gr (V.A.)

* Correspondence: arhchatz@uniwa.gr (A.C.); slalas@uth.gr (S.I.L.)

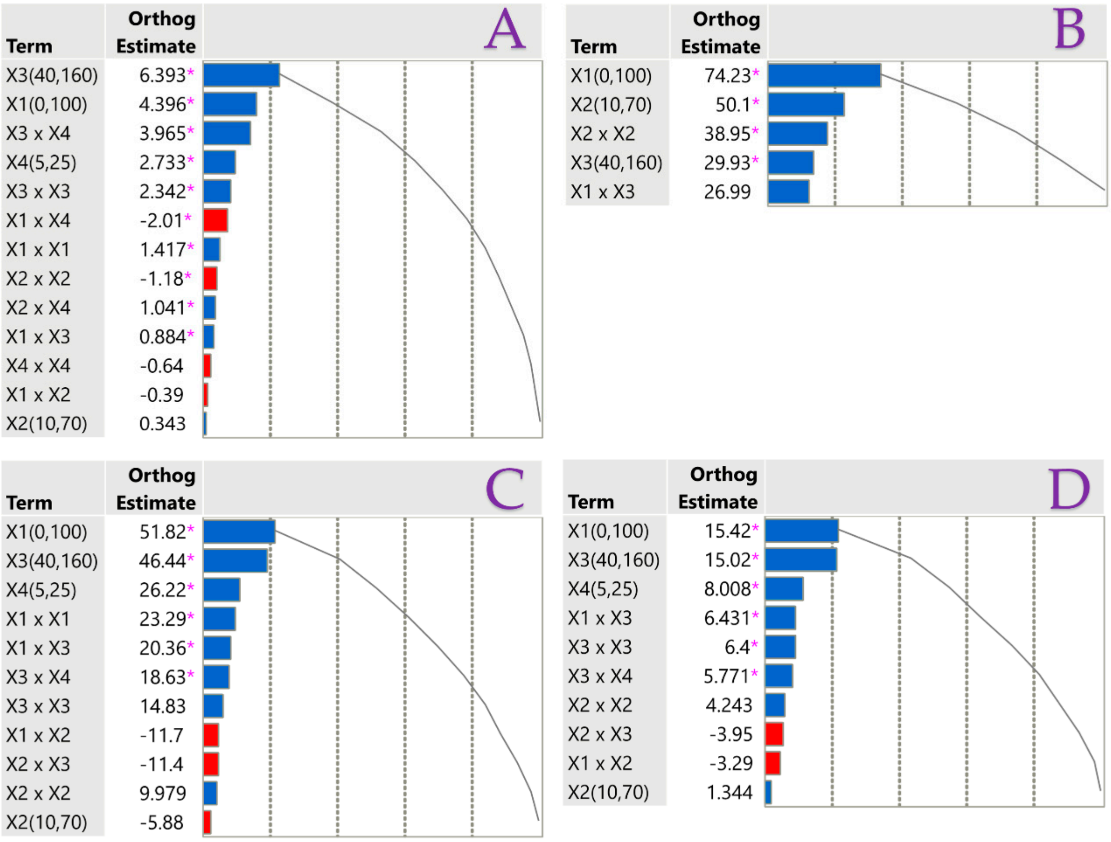

Figure S1. Pareto plots illustrating the significance of parameter estimates for the PLE technique across TPC (**A**), TAC (**B**), FRAP (**C**), and DPPH (**D**), with a pink asterisk marking significant values ($p < 0.05$). Positive estimates are shown in blue, while negative ones are represented in red.

Orthogonal estimates are widely used in regression and experimental design to evaluate the relative importance of individual factors while minimizing intercorrelation. This approach improves parameter estimation accuracy, reduces bias, and ensures that interactions among variables do not confound the calculated effects. In this study, a normalized Pareto plot was applied to assess the main effects and interactions of extraction parameters, with statistical

significance set at $p < 0.05$. For clarity, Figure S1 uses color coding: blue bars represent positive effects, whereas red bars indicate negative effects.

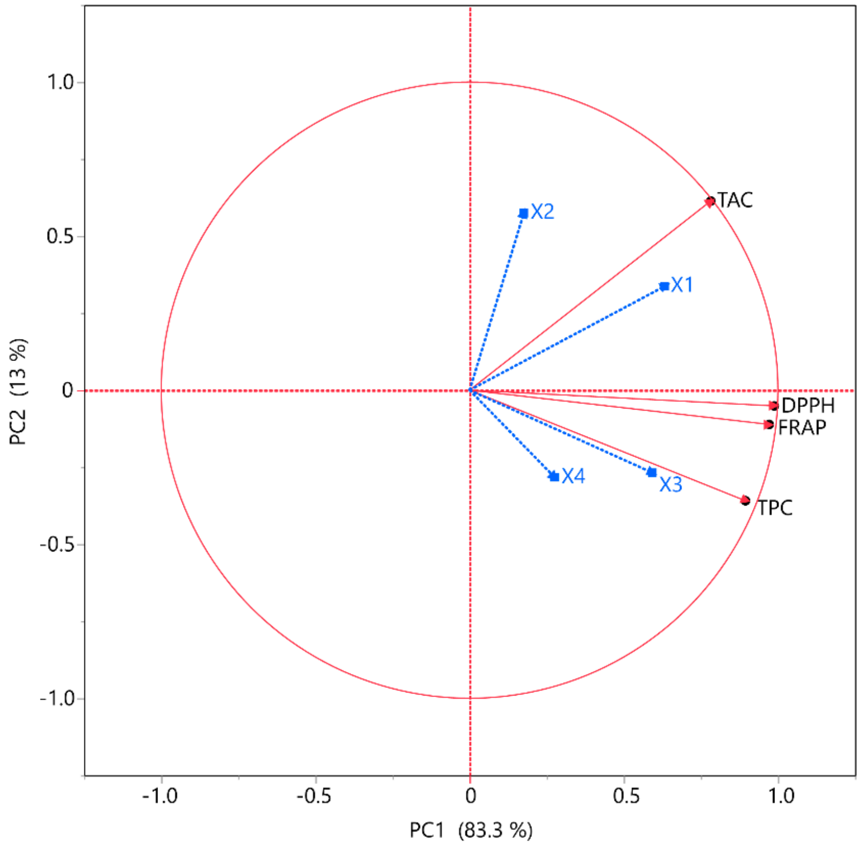

Figure S2. PCA for the measured variables. Each *X* variable is presented with a blue color.

Table S1. Pearson correlation coefficients among measured antioxidant responses.

Responses	TPC	TAC	FRAP	DPPH
TPC	-	0.5082	0.8562	0.8673
TAC		-	0.6703	0.7266
FRAP			-	0.988
DPPH				-

Principal component analysis (PCA) is an essential statistical method used for dimensionality reduction, allowing complex datasets to be simplified without losing critical information. By projecting the data onto a new coordinate system, PCA identifies directions of greatest variance, making it a powerful tool for exploratory analysis and data visualization. In this study, three technical replicates were included in the PCA to ensure result accuracy and minimize variability. This approach also helped confirm the consistency of the outcomes across multiple runs. The relationships among various bioactive compounds are presented in Figure S2 and Table S1, which show significant findings. As illustrated in Figure S2, the first principal component (PC1) explains 83.3% of the total variance and demonstrates a strong positive correlation with all assessed variables—TPC, TAC, FRAP, and DPPH—as well as with all extraction parameters: ethanol concentration (X_1), liquid-to-solid ratio (X_2), temperature (X_3), and extraction time (X_4).
